# The National Medical Union (Official)

**Published:** 1914-05-09

**Authors:** 


### THE NATIONAL MEDICAL UNION (Official).

Offices: 346 Strand. Tel.: City 8444.

### Meeting of Executive Committee.

A meeting of the Executive Committee was held at the offices of the Union on Saturday, May 2. Present: Dr. Greenyer, in the Chair; Drs. Brassey, Brierley, Oppenheimer, Porter, Greene, Johnston, Sharman, Carswell, and Mr. Dorrell.

In connection with the correspondence, the Hon. Secretaries were requested to intimate that, in accordance with the constitution of the Union as a federation of societies possessing local autonomy, the financial affairs of the local associations were entirely a matter for their own supervision, and that, in the event of the local subscriptions proving insufficient to meet local expenditure, a rise in the local rate was the only method of meeting the situation. So far as the Federation was concerned, receipt of a subscription of one guinea by either of the Honorary Treasurers constituted the sole claim to membership, entitling the subscriber to receive a weekly copy of The Hospital, in accordance with present arrangements, and to be present and vote as a member of the Union at the first General Meeting.

### The First General Meeting.

The Committee decided that it would be advisable to hold the first General Meeting of the Union at an early date, and Saturday, June 20, was fixed upon, subject to the approval of the Council. It was suggested that the meeting should begin at 10 a.m., with an afternoon session if necessary, and that the proceedings should end with a banquet. Provision was made for the formation of an Agenda Committee to draw up the report of the Provisional Council for presentation to the meeting, and also to prepare the agenda for the meeting. The Agenda Committee was also requested to prepare at the same time a prospectus of the Union for general distribution.

A temporary Parliamentary Committee was appointed, consisting of Dr. Greenyer, Dr. Sharman, Dr. Johnston, and Dr. Wilson, together with the Hon. Secretaries, to act in any emergency pending the formation of the permanent Parliamentary Committee as provided for in the constitution. The meeting terminated with an informal discussion on certain matters of policy which were relegated by the Council, at its last meeting, on March 21, to a special sub-committee to be appointed for the purpose.

### R.C.S. in Ireland and Unqualified Practice.

On April 19 the Royal College of Surgeons in Ireland passed the following resolution in reference to the authority conferred by the National Insurance Commissioners upon Insurance Committees to permit the insured to make arrangements for their medical treatment with persons "other than duly qualified medical practitioners," and to obtain a contribution from the Insurance funds towards the cost of such treatment:—

"The College observes with regret that in Section 44 (2) of the National Health Insurance (Medical Benefit) Regulations, 1913, and the Memorandum issued in connection therewith, provision is made whereby insured persons who make their own arrangements for medical benefit under Section 15 (3) of the National Insurance Act may obtain treatment from non-qualified persons.

"Hitherto none but duly qualified medical practitioners have been employed, as such, in any public capacity; and the College deplores that, under an Act professing to promote the health of the nation, recognition should be given and provision made for the payment of public money to a class of persons who have not obtained a legal qualification to practise medicine, and concerning whose medical knowledge there exists no sort of guarantee."

This resolution is similar to one passed by the Royal College of Physicians of London, and no doubt other medical corporations will follow the example set by the National Medical Union so long ago as January 31, and protest against this attempt on the part of the Insurance Commissioners to legalise and endow what the State considers, in every other connection except Insurance, to be unqualified and illegal medical practice. It is noteworthy that, notwithstanding the authority conferred by the objectionable regulation, the West Ham Insurance Committee declined at their last meeting to permit five insured persons to make their own arrangements for medical attendance and treatment by herbalists. If any reader knows of instances where such permission has been granted for "own arrangements" with any variety of non-qualified persons, he is invited to communicate full particulars to the National Medical Union.

### Panel Doctors versus Panel Chemists.

The working of the Insurance Act, or what should, more correctly speaking, be described as the attempt to work it at Salford, has brought about a situation which, however deplorable it may be from an ethical point of view, is yet not without amusing features.

The panel chemists, through the medium of the Pharmaceutical Committee, accuse the panel doctors of depriving them of their profits under the Act by over-prescribing and prescribing too frequently; and the "floating sixpence" having disappeared, the chemists suggest that the unpaid balance due to them from the Insurance Committee, to meet which there is no more money, shall be paid to them and surcharged to the panel doctors. The latter have retaliated by preparing a report embodying representations said to be supported by the Panel Committee, who are opposed to the surcharging of the doctors. The chemists also have prepared a report, and it is at this interesting phase of a very pretty quarrel that we are told that the ponderous administrative machinery of the Act is to be set rolling in order to smooth away all the difficulties.

Threatened Strike of Industrial Insurance Agents.

From the North of England also comes the first rumbling of a storm which, should it break, is likely to have a paralysing effect upon the working of the Act.

At a meeting of some 500 industrial insurance agents held in the Central Hall, Manchester, on May 1, a resolution was adopted "which," says the *Manchester Guardian*, "may involve a strike of some 30,000 of them and their fellow-workers throughout the country at the end of June." It concerned the rate of remuneration for agents' work under the National Health Act. The demand is that all agents should receive 1s. 6d. per member per annum instead of 1s. or 1s. 2d. as at present. Three societies only had so far recognised the claim to the full increase. The meeting of agents approved of the recent advance to 1s. 6d. in several cases, and they resolved to recognise 1s. 6d. as the minimum rate at which they would continue to do the work. The statement was made that, failing the concession, the agents will refuse to collect cards at the end of June.

But that is not apparently to be their final demand, for the resolution further expressed that those present would not be satisfied until 2s. 6d. per member per annum was conceded, and it was demonstrated by the general secretary that the societies could pay this sum and still have a profit of 11d. per member. The turn of the screw is coming from quite a novel and unexpected quarter, and it will be interesting to watch the attitude of the newly formed Faculty of Insurance. If profits are going to be cut like this, the Insurance Act will not be worth looking at from the commercial point of view.

Panel Plaint from Glasgow.

It is not a far cry from Manchester to Glasgow, where the discontent among the panel doctors has manifested itself in the form of a letter from the local Medical and Panel Committees to the Scottish Insurance Commissioners. It opens with the expression of intense dissatisfaction at the protracted delay in adjusting the medical accounts of last year. To be reminded of the "chaos of the year" can hardly be pleasant reading for Commissioners; nor can the demand to know when balances amounting to 15 to 20 per cent. of the total sums due to practitioners are going to be paid.

The letter proceeds to complain bitterly that the allocation of those insured persons who have preferred to choose their own doctor has been suspended, and that, too, in face of the fact that "on the strength of that expectation many practitioners incurred financial liabilities they would not otherwise have undertaken."

But a climax is reached in the "amazement and dismay" aroused when it was learned that the Commissioners proposed to withdraw from the panel doctors' lists the names of some 43,000 insured persons in Glasgow whose medical cards have been returned undelivered. This proceeding is "unfair," say the panel doctors of Glasgow, "to the insured." The alleged reason is a curious one. Because "*its immediate effect would be to deprive those people of their right to medical attendance and treatment*"!

### SANATORIUM BENEFIT STATISTICS.

A return issued on May 1 contains some very interesting statistics concerning the administration of sanatorium benefit under the Insurance Acts during the period from July 15, 1913, to January 11, 1914. It is a little difficult to base on the figures contained in the report accurate deductions as to the relative effects of the three kinds of "sanatorium benefit"—namely, residential treatment in a sanatorium, treatment at a tuberculosis dispensary, and domiciliary treatment. One reason for this difficulty is the fact that the cases contained in these three groups were doubtless of varying grades of severity; so that a mere comparison of the recovery rates does not afford a fair indication of the relative efficacy of the three methods of treatment. Another reason is that in a certain number of cases the same patient has been included in more than one category during the six months, so that the total got by adding the three lists together exceeds the actual number of individuals under treatment.

The figures for the dispensary cases are much less favourable than those for residential sanatorium treatment. Whether this merely reflects the superiority of institutional treatment for consumption, or is partly or wholly due to a selection of cases adverse to the statistics obtainable at the dispensaries, the report does not show. The similar figures for domiciliary treatment are not given in the abstracts which have been made public in the press: it is probable that they are much more depressing; and, if that is so, we are prepared to acquiesce in the policy of concealing them from public view.

Meanwhile, complaints are rife in many different quarters of the kingdom concerning the dearth of sanatorium accommodation for the insured, the delay which those in need of it have to experience, and the unsatisfactory nature of many of the buildings which have in several places been made to do duty as consumption sanatoria. At Macclesfield a typical instance of mismanagement in this respect has been provided. A ramshackle corrugated tin building, hastily erected during a smallpox scare ten years ago, has been converted into an Insurance Act "sanatorium." On one side is a bog, on another a fever hospital. The building itself is about as unlike a real sanatorium as it possibly could be, as anyone may satisfy himself by reading the description and photographs of it published in the *Manchester Courier* of April 9. We do not hesitate to describe this so-called building as a disgrace to the Act and to the Insurance Commission for permitting its use.

